# The Evolution of “Wipeout Wednesdays”: A Clinical Staff Engagement Initiative to Reduce CLABSIs in the NICU

**DOI:** 10.1097/pq9.0000000000000760

**Published:** 2024-09-25

**Authors:** Ilyse Richman, Lina Puntervold, Doron J. Kahn

**Affiliations:** * Neonatal Intensive Care Unit, Joe DiMaggio Children’s Hospital, Hollywood, Fla.; † Infection Control, Joe DiMaggio Children’s Hospital, Hollywood, Fla.; ‡ Division of Neonatology, Joe DiMaggio Children’s Hospital, Hollywood, Fla.; § Pediatrix Medical Group, Inc., Sunrise, Fla.

## Background:

The Wasie neonatal intensive care unit (NICU) at Joe DiMaggio Children’s Hospital is an 84-bed level IV NICU, caring for the most vulnerable neonates. Our unit has approximately 250 staff members, 50–60 of whom work during each shift. Although our central line blood stream infection (CLABSI) rate was low in 2020–2021 during COVID-19, in the first 6 months of 2022, we had as many CLABSIs as the entire previous year. We have had a CLABSI reduction project in place for many years; however, this rise in CLABSI rate necessitated new initiatives.

## Objectives:

To reduce our CLABSI standard infection ratio (SIR) by 20% from 0.8 to 0.64 by December 31, 2023. To realize this goal, a staff engagement initiative was implemented to (1) identify the main contributing factors for the increase in CLABSIs, (2) mutually create mitigation strategies for the identified factors, and (3) create plan-do-study-act cycles to test and implement new practices.

## Methods:

**Fig. 1.**
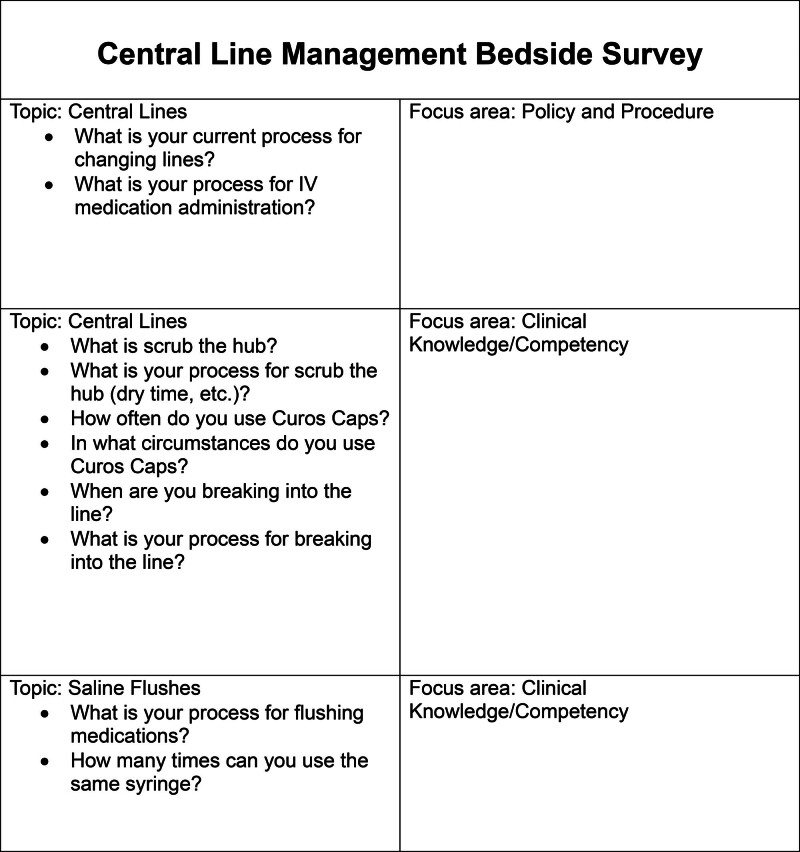
Central line bedside survey.

**Fig. 2.**
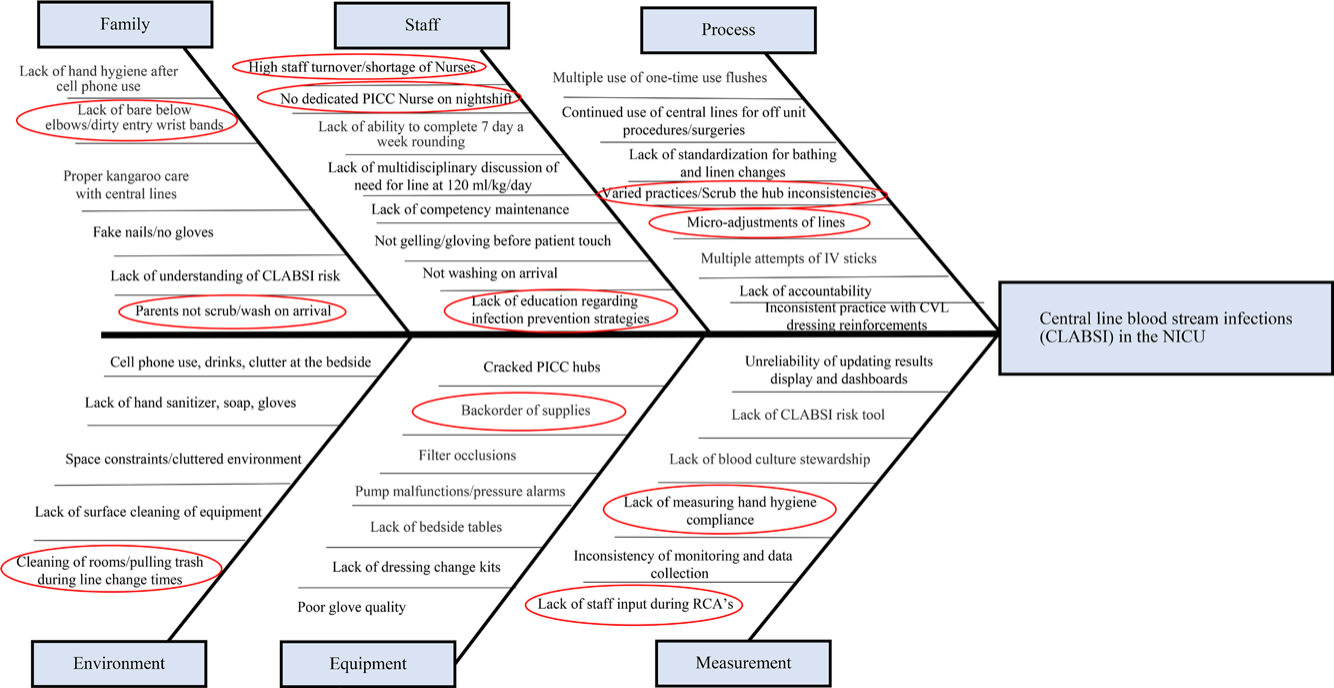
Staff-driven fishbone diagram. CVL, central venous line; PICC, peripherally inserted central catheter; RCA; root cause analysis.

Leadership performed an informal bedside survey (Fig. 1) focusing on the management of central lines. Realizing the importance of a multidisciplinary approach to achieve a global impact, nursing and physician NICU leadership, physicians and advanced practice providers, clinical nurses, vascular access nurses, ancillary personnel, environmental services, pharmacy, nursing educators, and patient and family-centered care advisory members were invited to meet, inspiring the concept of “Wipeout Wednesdays.” The initial Wipeout Wednesday meeting occurred on September 21, 2022, both in-person and virtual. The quality nurse specialist and infection preventionist reviewed the staff survey responses, data trends, and possible contributing factors. Based on extensive discussions with the team, a fishbone diagram (Fig. 2) and key driver diagram was created to help identify what we believed to be the key contributing and mitigating factors affecting our CLABSI rate. High staff turnover and novice staff members, inconsistent scrub the hub practices, supply backorders, handwashing noncompliance, and lack of education regarding infection prevention strategies were identified as some of the contributing factors. Over the course of 12 months, discussions at biweekly meetings, each of which we considered its own mini-plan-do-study-act cycle, presented new findings and opportunities for improvement.

## Results:

The initial meeting had 23 participants and participation continued to grow steadily until there was a sustained 50 members at each meeting for the final 6 months of 2023. Through multiple Wipeout Wednesdays, the participants were instrumental in creating an infection prevention parent contract, integration of Solutions for Patient Safety bundle competencies, implementation of weekly bedside central line rounds, establishment of staff-driven CLABSI root cause analyses, partnership with central supply for product needs, and the development of a back-to-basics education campaign including a unit “scrub the hub” song for engagement purposes. The ultimate results were a decrease in our CLABSI SIR by 27% to 0.58, exceeding our goal.

## Conclusion:

Through a combination of data transparency, multidisciplinary front-line engagement, leadership support to help remove barriers, and countermeasures in parallel with a staff-driven CLABSI prevention fishbone diagram and driver diagram, our team was able to exceed our goal of reducing our CLABSI SIR.

